# Extended inverse Lindley distribution: properties and application

**DOI:** 10.1186/s40064-015-1489-2

**Published:** 2015-11-10

**Authors:** Said Hofan Alkarni

**Affiliations:** Department of Quantitative Analysis, King Saud University, Riyadh, Saudi Arabia

**Keywords:** Inverse Lindley distribution, Extended inverse Lindley distribution, Upside-down bathtub lifetime data

## Abstract

In this paper, we introduce an extension of the inverse Lindley distribution, which offers more flexibility in modeling upside-down bathtub lifetime data. Some statistical properties of the proposed distribution are explicitly derived. These include density and hazard rate functions with their behavior, moments, moment generating function, skewness, kurtosis measures, and quantile function. Maximum likelihood estimation of the parameters and their estimated asymptotic distribution and confidence intervals are derived. Rényi entropy as a measure of the uncertainty in the model is derived. The application of the model to a real data set i.e., the flood levels for the Susquehanna river at Harrisburg, Pennsylvania, over 20 four-year periods from 1890 to 1969 is compared to the fit attained by some other well-known existing distributions.

## Background

Survival and reliability analysis is a very important branch of statistics. It has many applications in many applied sciences, such as engineering, public health, actuarial science, biomedical studies, demography, and industrial reliability. The failure behavior of any system can be considered as a random variable due to the variations from one system to another resulting from the nature of the system. Therefore, it seems logical to find a statistical model for the failure of the system. In other applications, survival data are categorized by their hazard rate, e.g., the number of deaths per unit in a period of time. The modeling of survival data depends on the behavior of the hazard rate. The hazard rate may belong to the monotone (non-increasing and non-decreasing hazard rate) or non-monotone (bathtub and upside-down bathtub [UBT] or unimodal hazard rate). Several lifetime models have been suggested in statistics literature to model survival data. The Weibull distribution is one of the most popular and widely used models in life testing and reliability theory. Lindley (1958) suggested a one-parameter distribution as an alternative model for survival data. This model is known as Lindley distribution. However, we suggest that Weibull and Lindley distributions are restricted when data shows non-monotone hazard rate shapes, such as the unimodal hazard rate function (Almalki and Nadarajah 2014; Almalki and Yuan 2013).

There are several real applications where the data show the non-monotone shape for their hazard rate. For example, Langlands et al. (1997) studied the data of 3878 cases of breast carcinoma seen in Edinburgh from 1954 to 1964 and noticed that mortality was initially low in the first year, reaching a peak in the subsequent years, and then declining slowly. Another real problem was analyzed by Efron (1988) who, using head and neck cancer data, found the hazard rate initially increased, reached a maximum, and decreased before it finally stabilized due to therapy. The inverse versions of some existing probability distributions, such as inverse Weibull, inverse Gaussian, inverse gamma, and inverse Lindley, show non-monotone shapes for their hazard rates; hence, we were able to model a non-monotone shape data.

Erto and Rapone (1984) showed that the inverse Weibull distribution is a good fit for survival data, such as the time to breakdown of an insulating fluid subjected to the action of constant tension. The use of Inverse Weibull was comprehensively described by Murthy et al. (2004). Glen (2011) proposed the inverse gamma distribution as a lifetime model in the context of reliability and survival studies. Recently, a new upside-down bathtub-shaped hazard rate model for survival data analysis was proposed by Sharma et al. (2014) by using transmuted Rayleigh distribution. Sharma et al. (2015a) introduced the inverse Lindley distribution as a one parameter model for a stress-strength reliability model. Sharma et al. (2015b) generalized the inverse Lindley into a two parameter model called “the generalized inverse Lindley distribution.” Finally, a new reliability model of inverse gamma distribution referred to as “the generalized inverse gamma distribution” was proposed by Mead (2015), which includes the inverse exponential, inverse Rayleigh, inverse Weibull, inverse gamma, inverse Chi square, and other inverse distributions.

The Lindley distribution was proposed by Lindley (1958) in the context of the Bayes theorem as a counter example of fiducial statistics with the probability density function (pdf)

1 $$f(y;\theta ) = \frac{{\theta^{2} }}{\theta + 1}(1 + y)e^{ - \theta y} ;\quad \theta ,y > 0.$$

Shanker et al. (2013) proposed two parameter extensions of the Lindley distribution with the pdf

2 $$f(z;\theta ) = \frac{{\theta^{2} }}{\theta + \beta }(1 + \beta z)e^{ - \theta z} ;\quad \theta ,\beta ,z > 0.$$

Ghitany et al. (2008) discussed the Lindley distribution and its applications extensively and showed that the Lindley distribution is a better fit than the exponential distribution based on the waiting time at the bank for service. The inverse Lindley distribution was proposed by Sharma et al. (2015a) using the transformation $X = \frac{1}{Y}$ with the pdf

$$f(x;\theta ) = \frac{{\theta^{2} }}{1 + \theta }\left( {\frac{1 + x}{{x^{3} }}} \right)e^{{ - \frac{\theta }{x}}} ;\quad \theta ,x > 0,$$

where $Y$ is a random variable having pdf (1).

Another two parameter inverse Lindley distribution introduced by Sharma et al. (2015a), called “the generalized inverse Lindley distribution,” is a new statistical inverse model for upside-down bathtub survival data that uses the transformation $X = Y^{{ - \frac{1}{\alpha }}}$ with the pdf

$$f(x;\theta ,\alpha ) = \frac{{\alpha \theta^{2} }}{1 + \theta }\left( {\frac{{1 + x^{\alpha } }}{{x^{2\alpha + 1} }}} \right)e^{{ - \frac{\theta }{{x^{\alpha } }}}} ;\quad \theta ,\alpha ,x\text{ > }0,$$

with $Y$ being a random variable having pdf (1).

Using the transformation $X = Z^{{ - \frac{1}{\alpha }}}$, we introduce a more flexible distribution with three parameters called “extended inverse Lindley distribution”, (EIL) and this gives us a better fit for upside-down bathtub data.

The aim of this paper is to introduce a new inverse Lindley distribution with its mathematical properties. These include the shapes of the density and hazard rate functions, the moments, moment generating function and some associated measures, the quantile function, and stochastic orderings. Maximum likelihood estimation of the model parameters and their asymptotic standard distribution and confidence interval are derived. Rényi entropy as a measure of the uncertainty in the model is derived. Application of the model to a real data set is finally presented and compared to the fit attained by some other well-known distributions.

## The extended inverse Lindley distribution

An extended inverse Lindley distribution with parameters $\theta ,\beta$, and $\alpha$ is defined by its probability density function and cumulative distribution function according to the definition.

### **Definition**

Let $Z$ be a random variable having pdf (2), then the random variable $X = Z^{{ - \frac{1}{\alpha }}}$ is said to follow an EIL distribution with probability density function

3 $$f(x;\theta ,\beta ,\alpha ) = \frac{{\alpha \theta^{2} }}{\theta + \beta }\left[ {\frac{{\beta + x^{\alpha } }}{{x^{2\alpha + 1} }}} \right]e^{{ - \frac{\theta }{{x^{\alpha } }}}} ;\quad \theta ,\beta ,\alpha ,x\text{ > }0$$

and cumulative distribution function (cdf)

4 $$\text{f}(x;\theta ,\beta ,\alpha ) = \frac{{\alpha \theta^{2} }}{\theta + \beta }\left[ {\frac{{\beta + x^{\alpha } }}{{x^{2\alpha + 1} }}} \right]e^{{ - \frac{\theta }{{x^{\alpha } }}}} ;\quad \theta ,\beta ,\alpha ,x \text{ > }0$$

### *Remark*

The pdf (3) can be shown as a mixture of two distributions as follows:

$$\text{F}(x;\theta ,\beta ,\alpha ) = \text{p}\text{f}_{1} (x) + (1 - p)\text{f}_{2} (x)$$

where

$$p = \frac{\theta }{\theta + \beta },\text{ }f_{1} (x) = \frac{\alpha \theta }{{x^{\alpha + 1} }}e^{{ - \frac{\theta }{{x^{\alpha } }}}} ,x > 0 \, \text\,\quad \text {{and}} \quad \, f_{2} (x) = \frac{{\alpha \theta^{2} }}{{x^{2\alpha + 1} }}e^{{ - \frac{\theta }{{x^{\alpha } }}}} ,\,{x} > 0.$$

We see that the EPL is a two-component mixture of inverse Weibull distribution (with shape $\alpha$ and scale $\theta$), and a generalized inverse gamma distribution (with shape parameters $2,\alpha$ and scale $\theta$), with the mixing proportion $p = \theta /(\theta + \beta )$.

We use $X \sim EIL(\theta ,\beta ,\alpha )$ to denote the random variable that has EIL distribution with parameters $\theta ,\beta ,\alpha$ and the pdf and cdf in (3) and (4), respectively.

The derivative of $f(x)$ is obtained from (3) as

$$f^{\prime}(x) = \frac{{\alpha \theta^{2} }}{\theta + \beta }x^{ - 3\alpha - 2} e^{{ - \frac{\theta }{{x^{\alpha } }}}} \psi (x^{\alpha } ),\quad \, x > 0,$$

where

$$\psi (y) = ay^{2} + by + c, \,\quad y = x^{\alpha } ,$$

with

$$a = - (\alpha + 1), \quad \, b = \alpha \theta - \beta (2\alpha + 1),\quad \, c = \alpha \beta \theta .$$

Clearly, $f^{\prime}(x)$ and $\psi (y)$ have the same sign and $\psi (y)$ is a unimodal quadratic function that attains its maximum value at the point $y$ whenever $\psi (y) = 0$; hence, the mode of $f(x)$ is given by

$$x = \left( {\frac{{\alpha \theta - \beta (2\alpha + 1) + \sqrt {[\alpha \theta - \beta (2\alpha + 1)]^{2} + 4\alpha \theta \beta (\alpha + 1)} }}{2(\alpha + 1)}} \right)^{{\frac{1}{\alpha }}} .$$

**Fig. 1:**
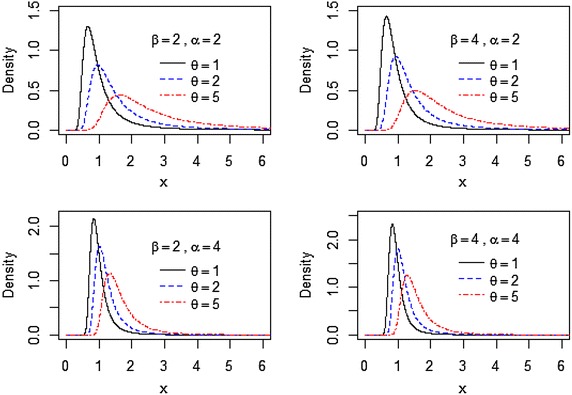
Plots of the probability density function of the EIL distribution for different values of $\theta ,\beta$, and $\alpha .$

In Fig. 1, we plot the pdf of the EIL distribution for some values of $\theta ,\beta ,\alpha$ and the behavior of $f(x)$.

## Survival and hazard functions

The survival and hazard rate functions of the EIL distribution are respectively given by

5 $$s(x) = 1 - F_{X} (x) = 1 - \left[ {1 + \frac{\theta \beta }{\theta + \beta }\frac{1}{{x^{\alpha } }}} \right]e^{{ - \frac{\theta }{{x^{\alpha } }}}} ;\quad \theta ,\beta ,\alpha ,x\text{ > }0,$$

and

6 $$h(x) = \frac{f(x)}{s(x)} = \frac{{\alpha \theta^{2} (\beta + x^{\alpha } )}}{{x^{\alpha + 1} \left[ {(\theta + \beta )x^{\alpha } \left( {e^{{\frac{\theta }{{x^{\alpha } }}}} - 1} \right) - \beta \theta } \right]}};\quad \theta ,\beta ,\alpha ,x\text{ > }0.$$

**Fig. 2:**
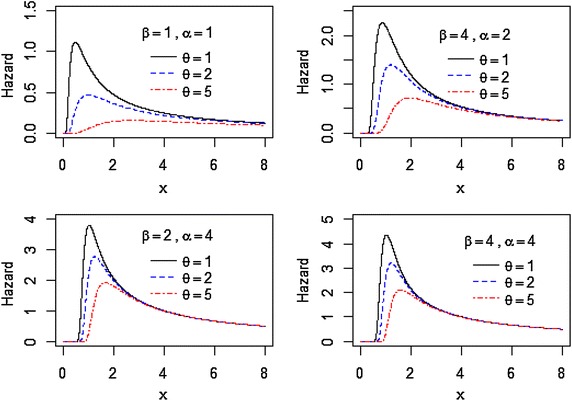
Plots of the hazard rate function of the EIL distribution for different values of $\theta ,\beta$, and $\alpha .$

The behavior of $h(x)$ in (6) of the $EIL(\theta ,\beta ,\alpha )$ for different values of the parameters $\theta ,\beta$, and $\alpha$ are showed graphically in Fig. 2.

## Moments, moment generating function, and associated measures

### **Theorem 1**

*Let*$X$*be a random variable that follows the EIL distribution with pdf as in* (3), *then the rth row moment (about the origin) is given by*

7 $$\mu_{r}^{{\prime }} = E(x^{r} ) = \frac{{\theta^{{\frac{r}{\alpha }}} [\alpha (\theta + \beta ) - r\beta ]}}{\alpha (\theta + \beta )}\Gamma \frac{\alpha - n}{\alpha },\quad \text{ }\alpha \text{ > }r,$$

*and the moment generating function (mgf) is givenby*

8 $$M_{X} (t) = \sum\limits_{n = 0}^{\infty } {\frac{{t^{n} }}{{n\text{!}}}} \text{ }\theta^{{\frac{n}{r}}} \frac{\alpha (\theta + \beta ) - n\beta }{\alpha (\theta + \beta )}\Gamma \frac{\alpha - n}{\alpha },\quad n\text{ < }\alpha ,$$

*where*$$\Gamma a = \int\limits_{0}^{\infty } {x^{a - 1} } e^{ - x} dx.$$

### *Proof*

$$\mu_{r}^{{\prime }} = E(x^{r} ) = \int\limits_{ - \infty }^{\infty } {x^{r} } f(x)dx$$

For $X \sim EIL(\theta ,\beta ,\alpha )$, we have

$$\begin{aligned} \mu_{r}^{{\prime }} = \frac{{\alpha \theta^{2} }}{\theta + \beta }\int\limits_{0}^{\infty } {x^{r} \left[ {\frac{{\beta + x^{\alpha } }}{{x^{2\alpha + 1} }}} \right]e^{{ - \frac{\theta }{{x^{\alpha } }}}} dx} \hfill \\ \text{ } = \frac{{\alpha \theta^{2} }}{\theta + \beta }\left[ {\beta \int\limits_{0}^{\infty } {\frac{{e^{{ - \frac{\theta }{{x^{\alpha } }}}} }}{{x^{2\alpha - r + 1} }}dx + \int\limits_{0}^{\infty } {\frac{{e^{{ - \frac{\theta }{{x^{\alpha } }}}} }}{{x^{\alpha - r + 1} }}dx} } } \right]. \hfill \\ \end{aligned}$$

Letting $y = x^{\alpha }$, we have

$$\mu_{r}^{{\prime }} = \frac{{\theta^{2} }}{\theta + \beta }\left[ {\beta \int\limits_{0}^{\infty } {\frac{{e^{{ - \frac{\theta }{y}}} }}{{y^{{3 - \frac{r}{\alpha }}} }}dy + \int\limits_{0}^{\infty } {\frac{{e^{{ - \frac{\theta }{y}}} }}{{y^{{2 - \frac{r}{\alpha }}} }}dy} } } \right].$$

Using $\int\limits_{0}^{\infty } {\frac{{e^{{ - \frac{a}{x}}} }}{{x^{b + 1} }}} dx = \frac{\Gamma b}{{a^{b} }},$ the definition of inverse gamma, the above expression is reduced to

$$\mu_{r}^{{\prime }} = \frac{{\theta^{{\frac{r}{\alpha }}} [\alpha (\theta + \beta ) - r\beta ]}}{\alpha (\theta + \beta )}\Gamma \frac{\alpha - n}{\alpha },\text{ }\alpha \text{ > }r.$$

The mgf of a continuous random variable $X,$ when it exists, is given by

$$M_{X} (t) = \int_{ - \infty }^{\infty } {e^{tx} f(x)dx.}$$

For $X \sim EIL(\theta ,\beta ,\alpha )$, we have

$$M_{X} (t) = \frac{{\alpha \theta^{2} }}{\theta + \beta }\int_{0}^{\infty } {e^{tx} \left[ {\frac{{\beta + x^{\alpha } }}{{x^{2\alpha + 1} }}} \right]e^{{ - \frac{\theta }{{x^{\alpha } }}}} dx.}$$

Using $e^{tx} = \sum\nolimits_{n = 0}^{\infty } {\frac{{t^{n} x^{n} }}{{n\text{!}}}} ,$ the series expansion, the above expression is reduced to

$$M_{X} (t) = \frac{{\alpha \theta^{2} }}{\theta + \beta }\sum\limits_{n = 0}^{\infty } {\frac{{t^{n} }}{{n\text{!}}}} \left[ {\beta \int\limits_{0}^{\infty } {\frac{{e^{{ - \frac{\theta }{{x^{\alpha } }}}} }}{{x^{2\alpha - n + 1} }}dx + \int\limits_{0}^{\infty } {\frac{{e^{{ - \frac{\theta }{{x^{\alpha } }}}} }}{{x^{\alpha - n + 1} }}dx} } } \right],$$

Letting $y = x^{\alpha }$, we have

$$M_{X} (t) = \frac{{\theta^{2} }}{\theta + \beta }\sum\limits_{n = 0}^{\infty } {\frac{{t^{n} }}{{n\text{!}}}} \left[ {\beta \int\limits_{0}^{\infty } {\frac{{e^{{ - \frac{\theta }{y}}} }}{{y^{{3 - \frac{n}{\alpha }}} }}dy + \int\limits_{0}^{\infty } {\frac{{e^{{ - \frac{\theta }{y}}} }}{{y^{{2 - \frac{n}{\alpha }}} }}dy} } } \right].$$

Using $\int\limits_{0}^{\infty } {\frac{{e^{{ - \frac{a}{x}}} }}{{x^{b + 1} }}} dx = \frac{\Gamma b}{{a^{b} }},$ the definition of inverse gamma, the moment generating function for the EIL distribution is given by

$$M_{X} (t) = \sum\limits_{n = 0}^{\infty } {\frac{{t^{n} }}{{n\text{!}}}} \theta^{{\frac{n}{r}}} \frac{\alpha (\theta + \beta ) - n\beta }{\alpha (\theta + \beta )}\Gamma \frac{\alpha - n}{\alpha },\quad n\text{ < }\alpha .$$

The mean and the variance of the EIL distribution are, respectively,

$$\begin{aligned} \mu = \frac{{\theta^{1/\alpha } (\alpha (\theta + \beta ) - \beta )}}{\alpha (\theta + \beta )}\Gamma \left( {\frac{\alpha - 1}{\alpha }} \right),\quad \text{ }\alpha \text{ > 1},\text{ } \hfill \\ \text{ }\sigma^{2} = \left[ {\frac{{\theta^{2/\alpha } }}{{\alpha^{2} (\beta + \theta )^{2} }}} \right]\left[ {\alpha (\beta + \theta )(\alpha (\beta + \theta ) - 2\beta )\Gamma \left( {\frac{\alpha - 2}{\alpha }} \right) - (\alpha (\beta + \theta ) - \beta )^{2} \Gamma^{2} \left( {\frac{\alpha - 1}{\alpha }} \right)} \right],\quad \alpha \text{ > 2}. \hfill \\ \end{aligned}$$

The $skewness{\text{ and }}kurtosis$ measures can be obtained from the expressions

$$\begin{aligned} skewness = \frac{{\mu_{3}^{{\prime }} - 3\mu_{2}^{{\prime }} \mu + 2\mu^{3} }}{{\sigma^{3} }} \hfill \\ curtosis = \frac{{\mu_{4}^{{\prime }} - 4\mu_{3}^{{\prime }} \mu + 6\mu_{2}^{{\prime }} \mu^{2} - 3\mu^{4} }}{{\sigma^{4} }}, \hfill \\ \end{aligned}$$

upon substituting for the row moments in (7).

## Quantile function

### **Theorem 2**

*Let*$X$*be a random variable with the pdf in* (3). *Then, the quantile function, say*$Q(p)$*is*

$$Q(p) = \left[ { - \frac{1}{\beta } - \frac{1}{\theta } - \frac{1}{\beta \theta }W_{ - 1} \left( { - \frac{p(\theta + \beta )}{{e^{(\theta + \beta )} }}} \right)} \right]^{{(\frac{ - 1}{\alpha })}} ,$$

*where*$\theta ,\beta ,\alpha \text{ > }0,\text{ }p \in (0,1)$, *and*$W_{ - 1} (.)$*is the negative Lambert*$W$*function*.

### *Proof*

We have $Q(p) = F^{ - 1} (p),\text{ }p \in (0,1)$, which implies $F(Q(p)) = p$. By substitution, we get

$$\left[ {1 + \frac{\theta }{\theta + \beta }\frac{\beta }{{(Q(p))^{\alpha } }}} \right]e^{{ - \frac{\theta }{{(Q(p))^{\alpha } }}}} = p.$$

When we multiply both sides by $- (\beta + \theta )e^{ - (\theta + \beta )}$, and raise them to $\beta$, we have the Lambert equation

$$- \left[ {\theta + \beta + \frac{\theta \beta }{{(Q(p))^{\alpha } }}} \right]e^{{ - \left( {\theta + \beta + \frac{\theta \beta }{{(Q(p))^{\alpha } }}} \right)}} = - p(\theta + \beta )e^{ - (\theta + \beta )} .$$

Hence, we have the negative Lambert $W$ function of the real argument $- p(\theta + \beta )e^{ - \theta - \beta }$. i.e.,

$$W_{ - 1} ( - p(\theta + \beta )e^{ - (\theta + \beta )} = - \left[ {\theta + \beta + \frac{\theta \beta }{{(Q(p))^{\alpha } }}} \right],$$

thus, by solving this equation for $Q(P)$, the proof is complete.

## Special cases of the EIL distribution

The EIL distribution contains some well-known distributions as sub-models, described below in brief.

### Inverse Lindley distribution

The inverse Lindley distribution (IL) shown by Sharma et al. (2015b) is a special case of the EIL distribution; $\alpha = \beta = 1.$ Using (3) and (4), the pdf and cdf is given by

$${\rm f}(x) = \frac{{\theta^{2} }}{1 + \theta }\left( {\frac{1 + x}{{x^{3} }}} \right)e^{{ - \frac{\theta }{x}}} ,\quad x\text{ > }0\quad \;\;{\text{and}}$$

$${\rm F}(x) = \left[ {1 + \frac{\theta }{1 + \theta }\frac{1}{x}} \right]e^{{ - \frac{\theta }{x}}} ,\quad x\text{ > }0.$$

The associated hazard rate function using (6) is given by

$${\rm h}(x) = \frac{{\theta^{2} (1 + x)}}{{x^{2} \left[ {(\theta + 1)x\left( {e^{{\frac{\theta }{x}}} - 1} \right) - \theta } \right]}},\quad x\text{ > }0.$$

### The generalized inverse Lindley distribution

The generalized inverse Lindley distribution (GIL) as shown by Sharma et al. (2015b) is a special case of the EIL distribution; $\beta = 1.$ Using (3) and (4), the pdf and cdf are respectively given by

$$f(x;\theta ,\alpha ) = \frac{{\alpha \theta^{2} }}{1 + \theta }\left( {\frac{{1 + x^{\alpha } }}{{x^{2\alpha + 1} }}} \right)e^{{ - \frac{\theta }{{x^{\alpha } }}}} ,\;\;{\text{and}}$$

$${\rm F}(x;\theta ,\alpha ) = \left[ {1 + \frac{\theta }{1 + \theta }\frac{1}{{x^{\alpha } }}} \right]e^{{ - \frac{\theta }{{x^{\alpha } }}}} .$$

The associated hazard rate function using (6) is given by

$${\rm h}(x) = \frac{{\alpha \theta^{2} (1 + x^{\alpha } )}}{{x^{\alpha + 1} \left[ {(1 + \beta )x^{\alpha } \left( {e^{{\frac{\theta }{{x^{\alpha } }}}} - 1} \right) - \theta } \right]}};\quad x\text{ > }0.$$

The rth row moment for the GIL is then given by

$$\mu_{r}^{{\prime }} = E(x^{r} ) = \frac{{\theta^{{\frac{r}{\alpha }}} [\alpha (\theta + 1) - r1]}}{\alpha (\theta + 1)}\Gamma \frac{\alpha - n}{\alpha },\quad \text{ }\alpha \text{ > }r,$$

and the mgf is given by

$$M_{X} (t) = \sum\limits_{n = 0}^{\infty } {\frac{{t^{n} }}{{n\text{!}}}} \theta^{{\frac{n}{r}}} \frac{\alpha (\theta + 1) - n}{\alpha (\theta + 1)}\Gamma \frac{\alpha - n}{\alpha },\quad n\text{ < }\alpha .$$

### Inverse Weibull distribution

The inverse Weibull distribution (IW) is a special case of EIL distribution; $\beta = 0$. Using (3) and (4), the pdf and cdf are respectively given by

$$f(x;\theta ,\alpha ) = \frac{\alpha \theta }{{x^{\alpha + 1} }}e^{{ - \frac{\theta }{{x^{\alpha } }}}} ,\quad x\text{ > }0\;\;{\text{and}}$$

$${\rm F}(x;\theta ,\alpha ) = e^{{ - \frac{\theta }{{x^{\alpha } }}}} ,\quad \text{ }x\text{ > }0.$$

The associated hazard rate function using (6) is given by

$${\rm h}(x) = \frac{\alpha \theta }{{x^{\alpha + 1} \left( {e^{{\frac{\theta }{{x^{\alpha } }}}} - 1} \right)}};\quad x\text{ > }0.$$

## Stochastic orderings

Stochastic orderings of positive continuous random variables is an important tool used judge comparative behavior. A random variable $X$ is said to be smaller than a random variable $Y$ in the following contexts:

- Stochastic order $$(X \le_{st} Y){\text{ if }}F_{X} (x) \le F_{Y} (x){ \forall }x;$$
- Hazard rate order $(X \le_{hr} Y){\text{ if }}h_{X} (x) \ge h_{Y} (x){ \forall }x;$
- Mean residual life order $(X \le_{mrl} Y){\text{ if }}m_{X} (x) \le m_{Y} (x){ \forall }x;$ and
- Likelihood ratio order $(X \le_{lr} Y){\text{ if }}f_{X} (x)/f_{Y} (x)\text{ }{\text{decreases in }}x.$

The following implications (Shaked and Shanthikumar 1994) are well known:

$$\begin{aligned} X \le_{lr} Y \Rightarrow X \le_{hr} Y \Rightarrow X \le_{mrl} Y \hfill \\ \text{ } \Downarrow \hfill \\ \text{ }X \le_{st} Y \hfill \\ \end{aligned}$$

The following theorem shows that the EIL distribution is ordered with respect to “likelihood ratio” ordering.

### **Theorem 3**

*Let*$$X \sim {\textit{PL}}(\theta_{1} ,\beta_{1,} \alpha_{1} )\;{\textit{and}}\;Y \sim {\textit{PL}}(\theta_{2} ,\beta_{2,} \alpha_{2} ).$$$${\textit{If}}\;\beta_{1} = \beta_{2}\; {\textit{and}}\; \theta_{2} \ge \theta_{1}\; {\textit{(or if}}\; \theta_{1} = \theta_{2} \; {\textit{and}} \; \beta_{2} \ge \beta_{1} ), \; {\textit{then}} \; X \ge_{lr} Y.\; {\textit{Hence,}}$$$$X \ge_{hr} Y,X \ge_{mrl} \;Y{\textit{and}}\; X \ge_{st} Y.$$

### *Proof*

We have

$$\frac{{f_{X} (x)}}{{f_{Y} (x)}} = \frac{{\alpha_{1} \theta_{1}^{2} }}{{\alpha_{2} \theta_{2}^{2} }}\frac{{\theta_{2} + \beta_{2} }}{{\theta_{1} + \beta_{1} }}\frac{{\beta_{1} + x^{{\alpha_{1} }} }}{{\beta_{2} + x^{{\alpha_{2} }} }}x^{{2(\alpha_{2} - \alpha_{1} )}} e^{{ - \theta_{2} x^{{ - \alpha_{2} }} - \theta_{1} x^{{ - \alpha_{1} }} }} ,$$

Setting $\alpha_{1} = \alpha_{2} = \alpha ,$ we have $\frac{{f_{X} (x)}}{{f_{Y} (x)}} = \frac{{\theta_{1}^{2} }}{{\theta_{2}^{2} }}\frac{{\theta_{2} + \beta_{2} }}{{\theta_{1} + \beta_{1} }}\frac{{\beta_{1} + x^{\alpha } }}{{\beta_{2} + x^{\alpha } }}e^{{(\theta_{2} - \theta_{1} )x^{ - \alpha } }}$, which is decreasing in $x$ for$\beta_{1} = \beta_{2} {\text{ and }}\theta_{2} \ge \theta_{1} {\text{ (or if }}\theta_{1} = \theta_{2} {\text{ and }}\beta_{2} \ge \beta_{1} ).$ This implies $X \le_{lr} Y$. Hence, $X \le_{hr} Y,X \le_{mrl} Y{\text{ and }}X \le_{st} Y.$

## Estimation and inference

Let $X_{1} , \ldots ,X_{n}$ be a random sample with observed values $x_{1} , \ldots ,x_{n}$ from EIL distribution. Let $\Theta = (\theta ,\beta ,\alpha )$ be the $3{ \times }1$ parameter vector. The log likelihood function is given by

$$\ln = n\,\log \alpha + 2\,n \text{log}\theta - n \text{log}(\theta + \beta )] + - (2\alpha + 1)\sum\limits_{i = 1}^{n} {\log x_{i} } - \theta \sum\limits_{i = 1}^{n} {x_{i}^{ - \alpha } } .$$

The score function $U_{n} (\Theta ) = ({\partial }\ln /\partial \theta ,{\partial }\ln /\partial \beta ,{\partial }\ln /\partial \alpha )^{T}$ is given by

$$\begin{aligned} \frac{{{\partial }\ln }}{\partial \theta } = \frac{2n}{\theta } - \frac{n}{\theta + \beta } - \sum\limits_{i = 1}^{n} {x_{i}^{ - \alpha } } , \hfill \\ \frac{{{\partial }\ln }}{\partial \beta } = \frac{ - n}{\theta + \beta } + \sum\limits_{i = 1}^{n} {\frac{1}{{1 + \beta x_{i}^{\alpha } }}} , \hfill \\ \frac{{{\partial }\ln }}{\partial \alpha } = \frac{n}{\alpha } + \sum\limits_{i = 1}^{n} {\frac{{x_{i}^{\alpha } \log x_{i} }}{{\beta + x_{i}^{\alpha } }}} - 2\sum\limits_{i = 1}^{n} {\log x_{i} } + \theta \sum\limits_{i = 1}^{n} {x_{i}^{ - \alpha } \log x_{i} } . \hfill \\ \end{aligned}$$

The maximum likelihood estimation (MLE) of $\Theta$ say $\{\Theta \}$ is obtained by solving the nonlinear system $U_{n} (\rm{x};\Theta ) = 0$. This nonlinear system of equations does not have a closed form. For interval estimation and hypothesis tests on the model parameters, we require the observed information matrix

$$I_{n} (\Theta ) = - \left[ {\begin{array}{*{20}c} {I_{\theta \theta } } & {I_{\theta \beta } } & {I_{\theta \alpha } } \\ {I_{\beta \theta } } & {I_{\beta \beta } } & {I_{\beta \alpha } } \\ {I_{\alpha \theta } } & {I_{\alpha \beta } } & {I_{\alpha \alpha } } \\ \end{array} } \right]$$

where the elements of $I_{n} \left( \varTheta \right)$ are the second partial derivatives of $U_{n} (\Theta )$. Under standard regular conditions for large sample approximation (Cox and Hinkley, 1974) that are fulfilled for the proposed model, the distribution of $\{\Theta \}$ is approximately $N_{3} (\Theta ,J_{n} (\Theta )^{ - 1} ),$ where $J_{n} (\Theta ) = E[I_{n} (\Theta )].$ Whenever the parameters are in the interior of the parameter space but not on the boundary, the asymptotic distribution of $\sqrt n (\{\Theta \} -\Theta )$ is $N_{3} (0,J(\Theta )^{ - 1} ),$ where $J(\Theta )^{ - 1} = \mathop {\lim }\limits_{n \to \infty } n^{ - 1} I_{n} (\Theta )$ is the unit information matrix and $p$ is the number of parameters of the distribution. The asymptotic multivariate normal $N_{3} (\Theta ,I_{n} (\{\Theta \})^{ - 1} )$ distribution of $\{\Theta \}$ can be used to approximate the confidence interval for the parameters, hazard rate, and survival functions. An $100 (1 - \gamma )$ asymptotic confidence interval for parameter $\Theta _{i}$ is given by

$$({\Theta }_{i} - Z_{{\frac{\gamma }{2}}} \sqrt {\widehat{{I^{ii} }}} ,{\Theta }_{i} + Z_{{\frac{\gamma }{2}}} \sqrt {\{{I^{ii} }\}} ),$$

where $\widehat{{I^{ii} }}$ is the $(i,i)$ diagonal element of $I_{n} (\{\Theta \})^{ - 1}$ for $i = 1, \ldots ,3$ and $Z_{{\frac{\gamma }{2}}}$ is the quantile $1 - \gamma /2$ of the standard normal distribution.

## Rényi entropy

Entropy is a measure of variation of the uncertainty in the distribution of any random variable. It provides important tools to indicate variety in distributions at particular moments in time and to analyze evolutionary processes over time. For a given probability distribution, Rényi (1961) gave an expression of the entropy function, so called Rényi entropy, defined by

$$\text{Re} (\gamma ) = \frac{1}{1 - \gamma }\log \left\{ {\int {f^{\gamma } (x)dx} } \right\}$$

where $\gamma \text{ > }0{\text{ and }}\gamma \ne 0.$ For EIL distribution in (3), we have

$$\begin{aligned} \text{Re} (\gamma ) = \frac{1}{1 - \gamma }\log \left\{ {\left( {\frac{{\alpha \theta^{2} }}{\theta + \beta }} \right)^{\gamma } \int\limits_{0}^{\infty } {\left[ {\frac{{\beta + x^{\alpha } }}{{x^{2\alpha + 1} }}} \right]^{\gamma } e^{{ - \frac{\gamma \theta }{{x^{\alpha } }}}} dx} } \right\} \hfill \\ \text{ } = \frac{1}{1 - \gamma }\log \left\{ {\left( {\frac{{\alpha \theta^{2} }}{\theta + \beta }} \right)^{\gamma } \int\limits_{0}^{\infty } {\left[ {\beta (1 + \frac{{x^{\alpha } }}{\beta })} \right]^{\gamma } x^{ - 2\gamma \alpha - \gamma } e^{{ - \frac{\gamma \theta }{{x^{\alpha } }}}} dx} } \right\}. \hfill \\ \end{aligned}$$

Now using the fact that $$(1 + z)^{\gamma } = \sum\limits_{j = 0}^{\infty } {\left( {\begin{array}{*{20}c} \gamma \\ j \\ \end{array} } \right)} z^{j} ,$$ we have

$$\text{Re} (\gamma ) = \frac{1}{1 - \gamma }\log \left\{ {\left( {\frac{{\alpha \theta^{2} }}{\theta + \beta }} \right)^{\gamma } \sum\limits_{j = 0}^{\infty } {\left( {\begin{array}{*{20}c} \gamma \\ j \\ \end{array} } \right)} \beta^{\gamma - j} \int\limits_{0}^{\infty } {\frac{{e^{{ - \frac{\gamma \theta }{{x^{\alpha } }}}} }}{{x^{\gamma (2\alpha - 1) - \alpha j} }}dx} } \right\}.$$

We substitute $y = x^{\alpha }$ and use the $\int\limits_{0}^{\infty } {\frac{{e^{{ - \frac{a}{x}}} }}{{x^{b + 1} }}} dx = \frac{\Gamma b}{{a^{b} }}$ definition of inverse gamma so that

$$\text{Re} (\gamma ) = \frac{1}{1 - \gamma }\log \left\{ {\left( {\frac{{\alpha \beta \theta^{2} }}{\theta + \beta }} \right)^{\gamma } \frac{1}{{\alpha (\gamma \theta )^{{\frac{\gamma }{\alpha }(2\alpha - 1) - \frac{1}{\alpha }}} }}\sum\limits_{j = 0}^{\infty } {\left( {\begin{array}{*{20}c} \gamma \\ j \\ \end{array} } \right)} \left( {\frac{\gamma \theta }{\beta }} \right)^{j} \Gamma [\frac{\gamma }{\alpha }(2\alpha - 1) - j - \frac{1}{\alpha }]} \right\},$$

where $$\Gamma a = \int\limits_{0}^{\infty } {x^{a - 1} } e^{ - x} dx.$$

## Application

In this section, we demonstrate the applicability of the EIL model for a real data. The data listed in Table 1 represents the flood levels for the Susquehanna River at Harrisburg, Pennsylvania, over 20 four-year periods from 1890 to 1969 and was obtained in a civil engineering context and give the maximum flood level (in millions of cubic feet per second). This data have been widely used by authors and were initially reported by Dumonceaux and Antle (1973). Upadhyay and Peshwani (2003) applied a Bayesian analysis for model comparison between lognormal and Weibull models and concluded that the lognormal fit the data better than the Weibull model. Singh et al. (2013) reported that inverse Weibull distribution fits this data better than other distributions, such as gamma, Weibull, flexible Weibull, and lognormal.

**Table 1:** Flood level data for the Susquehanna river

0.654	0.613	0.315	0.449	0.297
0.402	0.379	0.423	0.379	0.324
0.269	0.740	0.418	0.412	0.494
0.416	0.338	0.392	0.484	0.265

For this data, we fit the proposed $EIL(\theta ,\beta ,\alpha )$, the sub models that were introduced in “Special cases of the EIL distribution” and the three parameters generalized inverse Weibull proposed by De Gusmao et al. (2011), as well as.

**Fig. 3:**
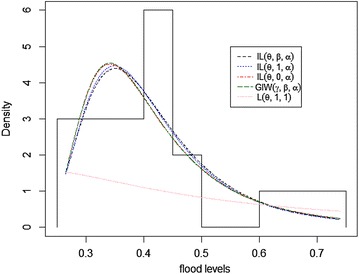
Plot of the fitted densities of the data in Table 1

**Fig. 4:**
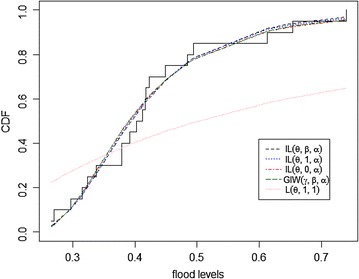
Plot of the fitted CDFs for the data in Table 1

The expectation–maximization (EM) algorithm is used to estimate the model parameters. The MLEs of the parameters, the Kolmogorov‒Smirnov statistics (K–S) with its respective *p* value, and the maximized log likelihood (logL) for the above distributions as well as our proposed model are given in Table 2. They indicate that the EIL distribution (proposed model) fits the data better than the other distributions. The $EIL(\theta ,\beta ,\alpha )$ takes the smallest K-S test statistic value and the largest value of its corresponding p-value. In addition, it takes the largest log likelihood. The fitted densities and the empirical distribution versus the fitted cumulative distributions of all models for this data are shown in Figs. 3 and 4, respectively.

**Table 2:** Parameter estimates, KS statistic, P-value and logL of flood level data

Dist.	$\hat{\theta }$	$\hat{\beta }$	$\hat{\alpha }$	K-S	P-value	$\log L$
$EIL(\theta ,\beta ,\alpha )$	0.1052	4.0439	2.9573	0.1395	0.8311	1 6.2317
$EIL(\theta ,1,\alpha )$	0.0899	–	3.0763	0.1445	0.7977	16.1475
$EIL(\theta ,0,\alpha )$	0.0123	–	4.2873	0.1545	0.7263	16.096
$GIW(\theta ,\beta ,\alpha )$	0.0302	4.3127	0.8071	0.1560	0.7150	1 6.097
$EIL(\theta ,1,1)$	0.6345	–	–	0.3556	0.0127	-0.5854

## Concluding remarks

In this paper, a new three-parameter inverse distribution, called extended inverse Lindley distribution, was introduced and studied in detail. This model has more flexibility than other types of inverse distributions (one, two and three parameters) due to the shape of its density as well as its hazard rate functions. It was shown that the density of the new distribution can be expressed as two components of the Weibull density function and a generalized gamma density function. We introduced the pdf, cdf, hazard rate function, the moments, moment generating function, and the quantile function in simple mathematical forms. Maximum likelihood estimation of the model parameters and their asymptotic standard distribution and confidence interval are derived. Rényi entropy as a measure of the uncertainty in the model is derived. Application of the model to a real data set is presented and compared to the fit attained by some other well-known inverse Lindley and inverse Weibull distributions, such as inverse Lindley, generalized inverse Lindley, inverse Weibull and generalized inverse Weibull.
